# Detection and Elimination of Senescent Cells with a Self-Assembled Senescence-Associated β-Galactosidase-Activatable Nanophotosensitizer

**DOI:** 10.1021/acs.jmedchem.3c01306

**Published:** 2023-12-19

**Authors:** Jacky C. H. Chu, Junlong Xiong, Clarence T. T. Wong, Shuai Wang, Dick Yan Tam, Alba García-Fernández, Ramón Martínez-Máñez, Dennis K. P. Ng

**Affiliations:** † Department of Chemistry, The Chinese University of Hong Kong, Shatin, N.T., Hong Kong, China; ‡ Department of Pharmacy, The Affiliated Luohu Hospital of Shenzhen University, Shenzhen University, Shenzhen 518001, China; § Department of Applied Biology and Chemical Technology, The Hong Kong Polytechnic University, Kowloon, Hong Kong, China; ∥ Instituto Interuniversitario de Investigación de Reconocimiento, Molecular y Desarrollo Tecnológico, Universitat Politècnica de València, Universitat de València, Valencia46022, Spain; ⊥ CIBER de Bioingeniería, Biomateriales y Nanomedicina, Instituto de Salud Carlos III, Madrid 28029, Spain; # Unidad Mixta UPV-CIPF de Investigación en Mecanismos de Enfermedades y Nanomedicina, Centro de Investigación Príncipe Felipe, Universitat Politècnica de València, Valencia46012, Spain; ∇ Unidad Mixta de Investigación en Nanomedicina y Sensores, Instituto de Investigación Sanitaria La Fe (IIS La Fe), Universitat Politècnica e València, Valencia 46026, Spain

## Abstract

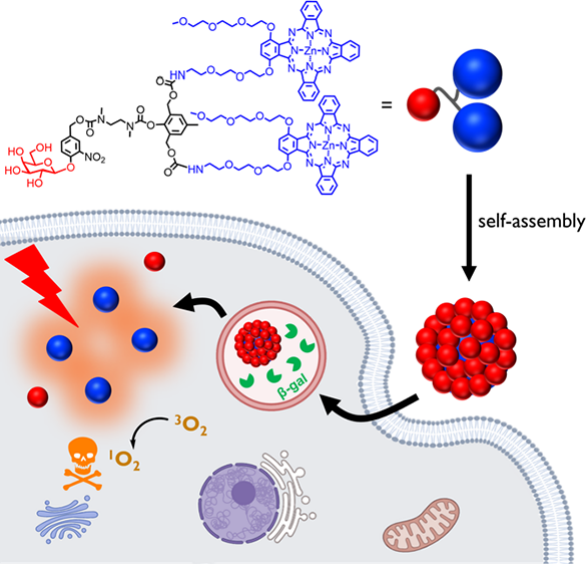

Senescent cells have become an important therapeutic target for many age-related dysfunctions and diseases. We report herein a novel nanophotosensitizing system that is responsive to the senescence-associated β-galactosidase (β-gal) for selective detection and elimination of these cells. It involves a dimeric zinc(II) phthalocyanine linked to a β-galactose unit via a self-immolative linker. This compound can self-assemble in aqueous media, forming stable nanoscale particles in which the phthalocyanine units are stacked and self-quenched for fluorescence emission and singlet oxygen production. Upon internalization into senescent HeLa cells, these nanoparticles interact with the overproduced senescence-associated β-gal inside the cells to trigger the disassembly process through enzymatic cleavage of the glycosidic bonds, followed by self-immolation to release the photoactive monomeric phthalocyanine units. These senescent cells can then be lit up with fluorescence and eliminated through the photodynamic action upon light irradiation with a half-maximal inhibitory concentration of 0.06 μM.

## Introduction

Cellular senescence is a stereotypical state of cessation of cell division happening in response to stress-induced cellular damage.^1,2^ It has both physiological and tissue remodeling roles during development and after injury to maintain tissue homeostasis and suppress tumor growth.^3^ However, senescent cells tend to accumulate in tissues and promote the release of various inflammatory cytokines, chemokines, and matrix remodeling factors, which results in inflammation, tissue aging, and destruction. In fact, cellular senescence is related to multiple pathologies of aging and is closely associated with a range of age-related diseases such as type II diabetes, cancer, and neurodegenerative disorders.^4−6^ Therefore, cellular senescence has emerged as an important therapeutic target for aging-related disorders,^7,8^ and selective detection and elimination of senescent cells are of great importance.

Senescent cells are characterized by various biomarkers, including epigenetic changes, activation of p53/p21^CIP^ and p16^INK4a^/pRB tumor-suppressor pathways, mitochondrial dysfunction, a senescence-associated secretory phenotype, and upregulation of senescence-associated β-galactosidase (β-gal) in the lysosomes.^9,10^ The last one, in particular, is probably the most common biomarker used for characterizing cellular senescence. Over the past decade, various bioanalytical methods have been developed for the detection of cellular senescence,^11−13^ and a number of senolytic strategies have also been reported for the removal of senescent cells.^14,15^ In particular, the senolytic agents dasatinib and quercetin have already entered different phases of clinical trials. However, these first-generation drugs generally lack high specificity toward senescent cells, which inevitably causes off-target toxicities and limits their clinical use.^16^

To improve the therapeutic efficacy of senolytics, a range of nanomaterials have been used as carriers, some of which are responsive toward β-gal overexpressed in senescent cells.^17−19^ Such delivery systems generally exhibit improved bioavailability and higher stability compared to molecular drugs. Besides, they can also facilitate the targeted delivery and controlled release of senolytic agents to senescent cells and reduce their adverse effects. For example, mesoporous silica nanoparticles coated with galacto-oligosaccharides have been used to encapsulate fluorophores, cytotoxic drugs, and senolytic agents, which can be released preferentially in senescent cells and tumor-bearing mice with senescence-inducing chemotherapy.^20,21^ Other strategies, such as the use of β-2-microglobulin^22^ or CD9^23^ monoclonal antibody on the surface of nanoparticles to recognize senescent cells, followed by the clearance or attenuation of these cells by the encapsulated therapeutic agents, have also been reported. However, despite advances in the development of nanosenolytics in recent years, there is still a strong demand for effective theranostic agents that can selectively detect and eliminate senescent cells.

As an innovative anticancer modality, photodynamic therapy (PDT) has attracted increasing attention.^24,25^ It involves light irradiation on a tumor in which a photosensitive drug has accumulated to trigger the interactions with the endogenous oxygen to produce highly cytotoxic reactive oxygen species (ROS) that result in tumor eradication. Owing to the unique mechanism, PDT is regarded as a noninvasive modality without the problem of drug resistance. The treatment outcome depends largely on the tumor specificity of the photosensitizers, the oxygen content in the tumor microenvironment, the extent of light penetration, the cell death pathways, etc.^26^ Recent advances aim to enhance the tumor specificity of the photodynamic action so as to prevent unwanted photodamage to normal cells and tissues. To this end, various approaches have been adopted, such as conjugation of the photosensitizers with a tumor-targeting ligand to promote the uptake by cancer cells, encapsulation of the photosensitizers into nanoparticles to enhance the tumor localization by the enhanced permeability and retention effect, and the development of smart photosensitizers that can be selectively activated by tumor-associated stimuli.^27−29^ With high versatility, PDT has also been clinically used for microbial infections in dentistry^30^ and the treatment of certain noncancerous conditions, such as acne vulgaris^31^ and polypoidal choroidal vasculopathy,^32^ and has a high potential for the elimination of senescent cells.

Over the years, while many fluorescent probes have been constructed for the detection of intracellular β-gal,^13,33,34^ only a handful of β-gal-activable photosensitizers have been reported. Nagano and co-workers and Urano and co-workers developed several photosensitizers based on thiazole orange,^35^ xanthene,^36^ or selenium-modified xanthene^37,38^ that could selectively eliminate β-gal-expressing HeLa and *lac*Z gene-transfected HEK293 cells. The examples were extended to an iodinated resorufin-based photosensitizer that was able to remove β-gal-overexpressing glioblastoma cells.^39^ It is worth noting that for all of these examples, the in vitro target was β-gal-overexpressing cancer cells, or *lac*Z gene-transfected cells instead of senescent cells. For the latter, the bacterial β-gal expression is in the cell cytoplasm, while the β-gal activity is in the lysosomes for senescent cells. In fact, to the best of our knowledge, only three molecular senescence-associated β-gal-activatable photosensitizers have been reported so far, which include a methylene blue derivative reported by Yang and co-workers^40^ and Tung and co-workers^41^ independently, a selenium-containing naphthoquinone methide reported by Li and co-workers,^42^ and a boron dipyrromethene (BODIPY) reported by us recently.^43^

Based on the above and following our interest in cellular senescence, we report herein the first example of nanophotosensitizers for the photodynamic elimination of senescent cells. The use of nanoparticles can promote the self-quenching of the encapsulated photosensitizing molecules in the native form and lead to a more remarkable activation effect upon stimulus-triggered dissociation in the target cells. Compared with the aforementioned molecular systems,^40−43^ this nanophotosensitizer exhibits much higher photocytotoxicity against the senescent cells.

## Results and Discussion

### Design and Preparation

Owing to their desired photophysical properties, high stability, and ease of chemical modification, zinc(II) phthalocyanines (ZnPcs) have served as efficient photosensitizers for PDT.^44^ Having a large hydrophobic π platform, molecules of these compounds tend to aggregate in aqueous media. The molecular stacking is exaggerated when the ZnPc units are connected covalently or encapsulated in nanoparticles, resulting in effective quenching of the fluorescence emission and ROS generation. This intrinsic property has been utilized to design activatable photosensitizers, both in molecular and nano forms, for which tumor-associated stimuli can trigger the release of free ZnPc units, thereby restoring their photoactivities.^45,46^ On this basis, we believed that the connection of two ZnPc units to a β-gal substrate via a self-immolative linker could give a self-quenched ZnPc dimer that would be responsive toward β-gal. According to our previous findings for dimeric ZnPcs, while the fluorescence emission can be largely quenched by self-quenching, their singlet oxygen generation cannot be inhibited effectively.^47,48^ As a result, the effect of activation on photocytotoxicity is not very significant. To remedy this problem, we envisaged that by encapsulating the molecules of the dimeric ZnPc in their self-assembled nanoparticles, it could promote the aggregation-induced quenching of the fluorescence emission and singlet oxygen generation^49^ and eventually lead to a more remarkable activation effect upon interaction with β-gal.

**Figure 1:**
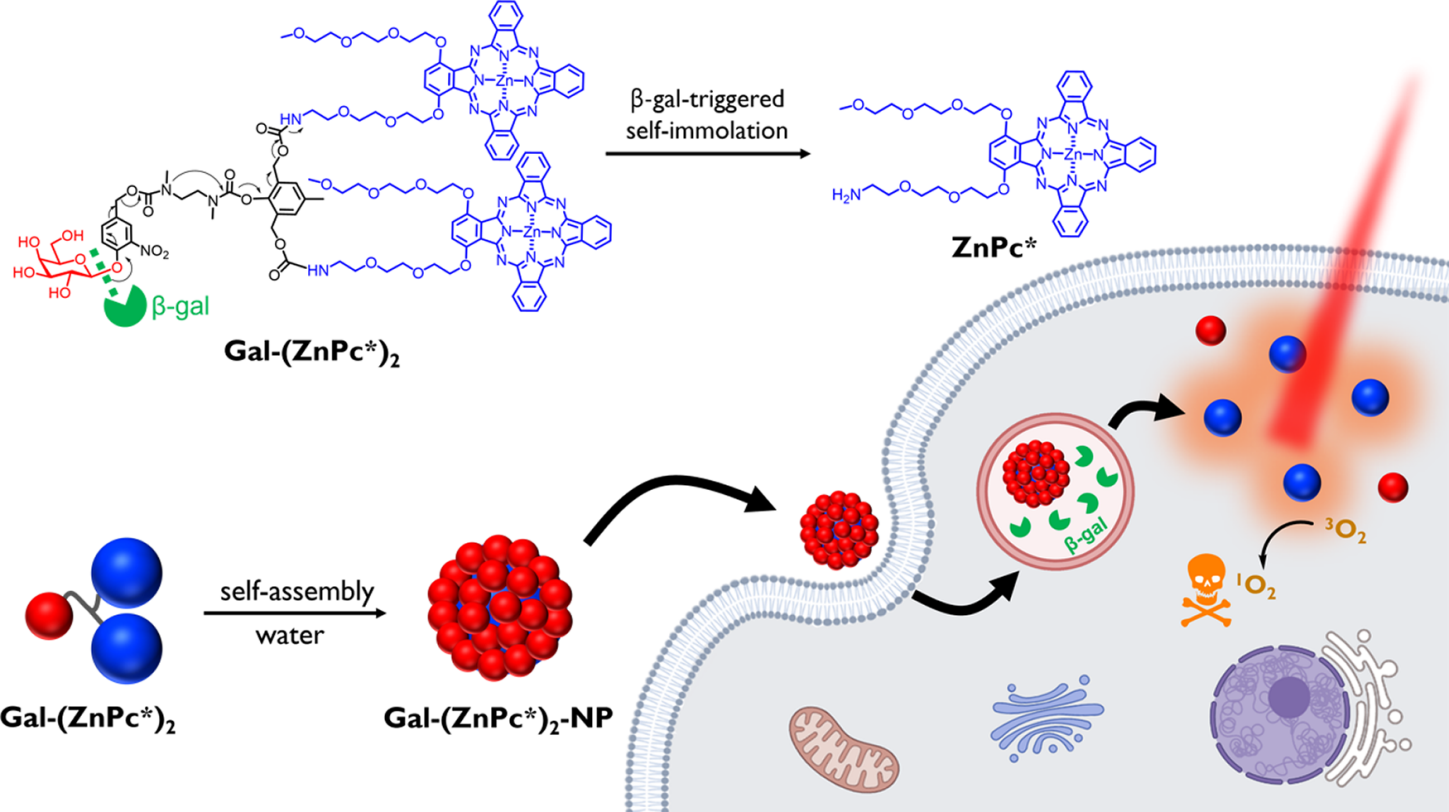
Schematic illustration of the mechanistic action of the senescence-associated β-gal-activatable nanophotosensitizing system for the detection and elimination of senescent cells.

Figure 1 illustrates the mechanistic action of this β-gal-activatable nanophotosensitizing system designed for both the detection and elimination of senescent cells. It involves β-galactose-conjugated dimeric ZnPc, labeled as Gal-(ZnPc*)_**2**_, which can undergo β-gal-triggered self-immolation to release two photodynamically active monomeric ZnPc* units. Having two hydrophobic phthalocyanine rings that are held by π–π interaction and several hydrophilic triethylene glycol and galactose moieties, this amphiphilic ZnPc dimer self-assembles in aqueous media to form nanoparticles, labeled as Gal-(ZnPc*)_**2**_**-NP**. Due to the strong π–π and hydrophobic interactions of the ZnPc moieties, the photoactivities of ZnPc in the nanoparticles are largely quenched in its native form. Upon internalization into senescent cells, the nanoparticles undergo disassembly and enzymatic cleavage of the glycosidic bonds by the overproduced senescence-associated β-gal, triggering the self-immolation and release of free ZnPc* units. Upon light irradiation, the fluorescence emission and ROS generation of ZnPc* are largely restored, enabling both fluorescence imaging and the photodynamic elimination of the senescent cells.

**Scheme 1:**
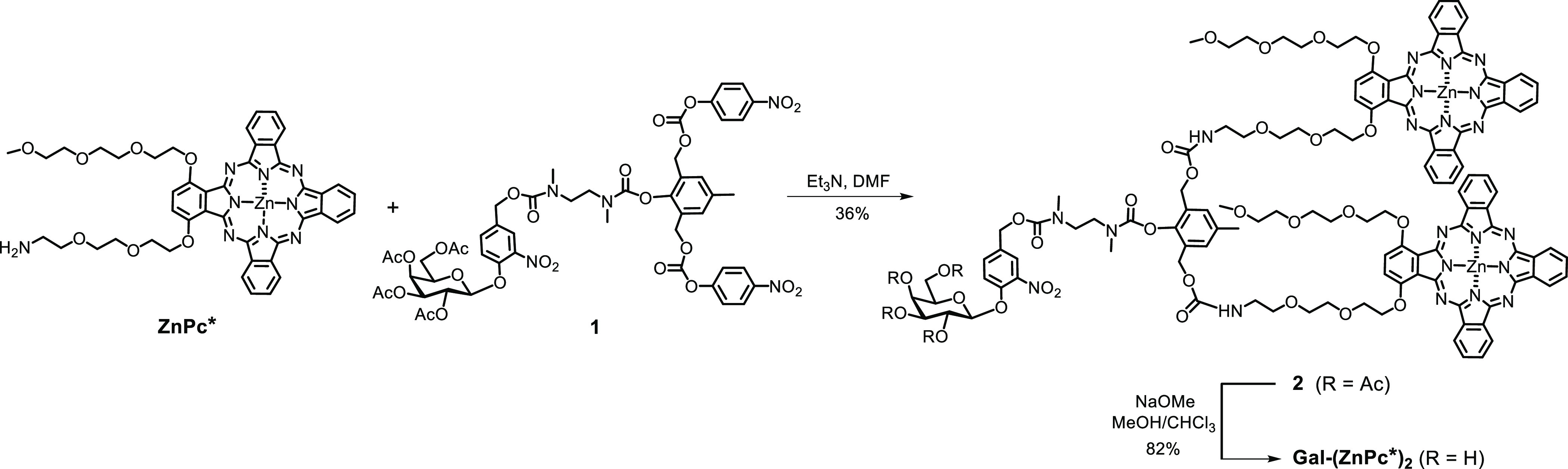
Synthetic Scheme of Gal-(ZnPc*)_**2**_

The β-gal-activatable Gal-(ZnPc*)_**2**_ was prepared by condensation of our previously reported ZnPc*(50) and the β-galactose-substituted AB_2_-type self-immolative linker **1**(43) in *N*,*N*-dimethylformamide (DMF), followed by hydrolysis of the intermediate product **2** to remove the acetyl groups (Scheme 1). ZnPc* is a versatile precursor, which contains a triethylene glycol chain to increase the water solubility of the phthalocyanine and promote its cellular uptake, as well as an amine-modified chain to facilitate further conjugation. This compound can be synthesized readily as a single isomer through the ″3 + 1″ mixed cyclization. Compound **1** contains a self-immolative AB_2_-type platform that can connect to various substrates and therapeutic components for controlled drug delivery.^51^ With a β-galactose terminal group, this compound is responsive toward β-gal and has been used by us previously for the construction of a β-gal-activatable photosensitizer.^43^ Both **2** and Gal-(ZnPc*)_**2**_ were characterized with ^1^H NMR spectroscopy and electrospray ionization (ESI) mass spectrometry. The purity of Gal-(ZnPc*)_**2**_ was determined to be >95% by reverse-phase high-performance liquid chromatography (HPLC) (Figures S1–S5 in Supporting Information).

**Figure 2:**
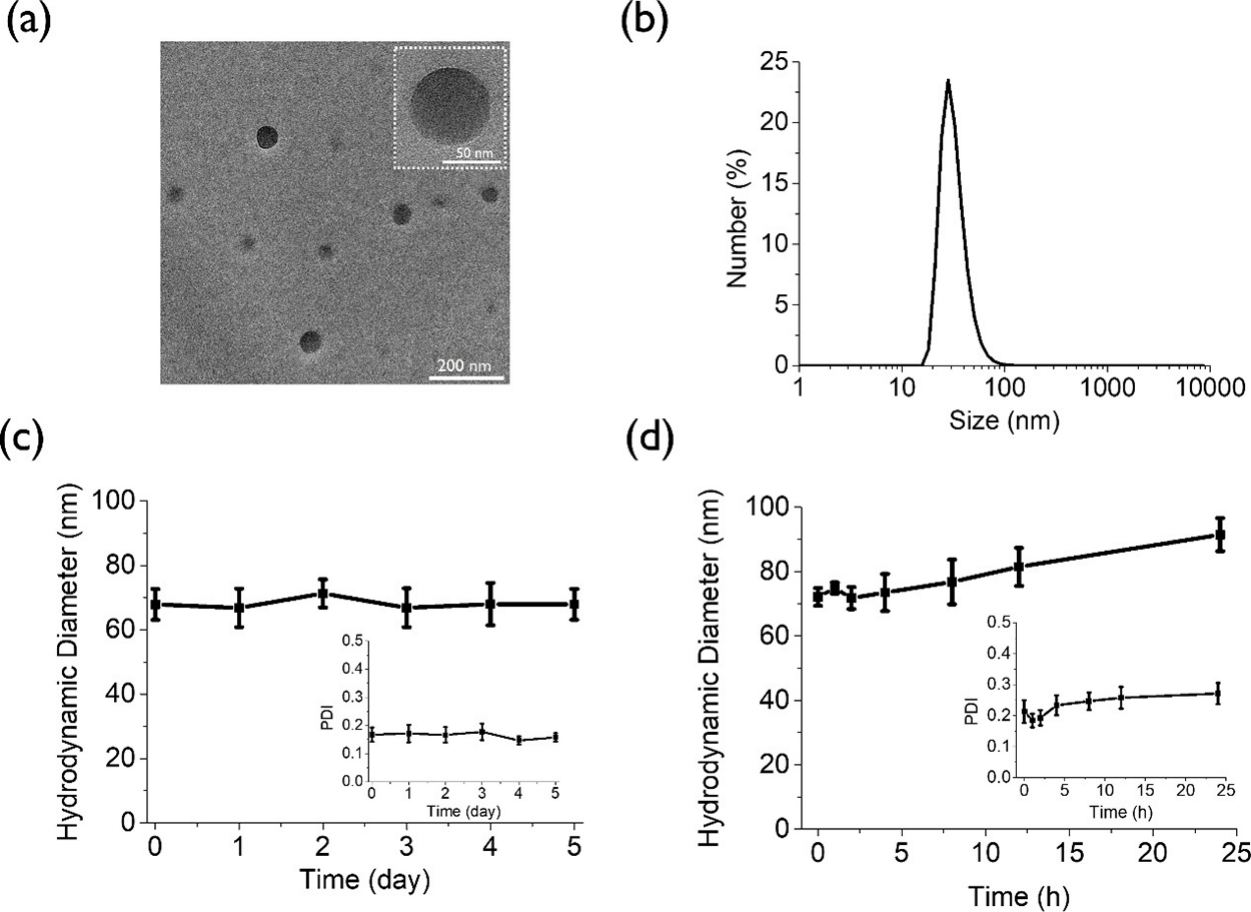
(a) TEM image of Gal-(ZnPc*)_**2**_**-NP**. (b) Hydrodynamic diameter distribution of Gal-(ZnPc*)_**2**_**-NP** in water. Change in hydrodynamic diameter and PDI (inset) of Gal-(ZnPc*)_**2**_**-NP** (c) in water over a period of 5 days and (d) in RPMI 1640 medium over a period of 24 h at room temperature.

To prepare the self-assembled nanosystem, Gal-(ZnPc*)_**2**_ was first dissolved in dimethyl sulfoxide (DMSO) to form a stock solution (0.4 mM). An aliquot of this solution (100 μL) was added slowly into water (3.9 mL), and then the mixture was sonicated for 2 h to afford the self-assembled nanoparticles Gal-(ZnPc*)_**2**_**-NP**. As characterized by transmission electron microscopy (TEM), they were spherical in shape with a size of about 70 nm (Figure 2a). By dynamic light scattering (DLS), the intensity-averaged hydrodynamic diameter of these nanoparticles was determined to be 67.9 ± 4.8 nm (Figure 2b) with a polydispersity index (PDI) of 0.17 ± 0.03. To study the stability of these nanoparticles, they were incubated in water and Roswell Park Memorial Institute (RPMI) 1640 medium, respectively, and then their size was monitored by DLS over a period of time. As displayed in Figure 2c, the hydrodynamic diameter of the nanoparticles was essentially unchanged in water over 5 days. In RPMI 1640 medium, the hydrodynamic diameter slightly increased from 72.1 ± 2.8 to 91.4 ± 5.2 nm over a period of 24 h (Figure 2d). The small increase in the size may be attributed to the binding of the nanoparticles with the proteins in the medium, which was also observed in our previously reported self-assembled phthalocyanine-based nanoparticles.^52^ The results showed that Gal-(ZnPc*)_**2**_**-NP** was stable in these aqueous media.

### β-Gal-responsive Spectroscopic and Photophysical Properties

**Figure 3:**
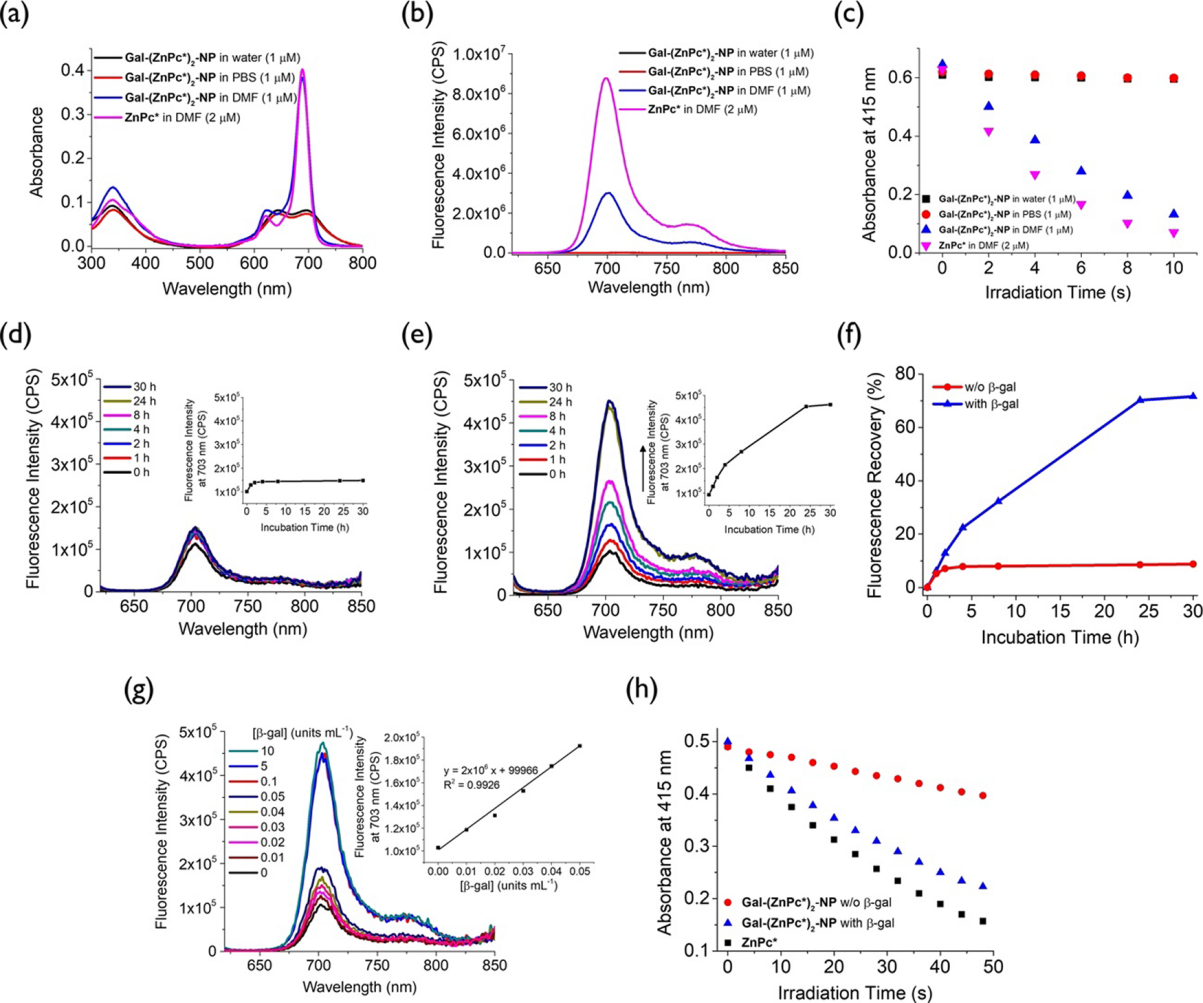
(a) Electronic absorption and (b) fluorescence (λ_ex_ = 610 nm) spectra of Gal-(ZnPc*)_**2**_**-NP** (1 μM) in water, PBS, and DMF, respectively, and ZnPc* (2 μM) in DMF. (c) Rates of consumption of DPBF (initial concentration = 30 μM) sensitized by Gal-(ZnPc*)_**2**_**-NP** (1 μM) in water, PBS, and DMF, respectively, and ZnPc* (2 μM) in DMF upon light irradiation (λ > 610 nm). Change in fluorescence spectrum of Gal-(ZnPc*)_**2**_**-NP** (1 μM) in the (d) absence and (e) presence of β-gal (10 unit mL^–1^) in PBS with Tween 80 (0.01% v/v) at 37 °C over a period of 30 h. The inset of each figure shows the change in fluorescence intensity at 703 nm with time. (f) Percentage of fluorescence recovery of Gal-(ZnPc*)_**2**_**-NP** (1 μM) in the absence and presence of β-gal (10 unit mL^–1^) in PBS with Tween 80 (0.01% v/v) at 37 °C over a period of 30 h. (g) Change in fluorescence spectrum of Gal-(ZnPc*)_**2**_**-NP** (1 μM) in PBS with Tween 80 (0.01% v/v) after mixing with different concentrations of β-gal at 37 °C for 30 h. The inset shows the change in fluorescence intensity at 703 nm with a concentration of β-gal from 0 to 0.05 unit mL^–1^. (h) Rates of consumption of DPBF (initial concentration = 30 μM) sensitized by Gal-(ZnPc*)_**2**_**-NP** (1 μM) with and without pretreatment with β-gal (10 unit mL^–1^) at 37 °C for 30 h and ZnPc* (2 μM) in PBS with Tween 80 (0.01% v/v) upon light irradiation (λ > 610 nm).

The electronic absorption and fluorescence spectra of Gal-(ZnPc*)_**2**_**-NP** (1 μM) were measured in water, phosphate-buffered saline (PBS), and DMF, respectively, and compared with those of monomeric ZnPc* (2 μM) (Figure 3a,b). The last solvent was expected to be able to disrupt the noncovalent interactions of the molecules, resulting in the disassembly of the nanoparticles to generate free Gal-(ZnPc*)_**2**_.^52^ As expected, the absorption spectrum of Gal-(ZnPc*)_**2**_**-NP** in DMF showed a strong Q-band at 689 nm, which was virtually the same as that of ZnPc*. However, its fluorescence emission at ca. 700 nm was approximately 3-fold weaker than that of ZnPc*, which could be attributed to the self-quenching of the dimeric system. In contrast, the Q-band of Gal-(ZnPc*)_**2**_**-NP** in water or PBS was significantly broadened and weakened, and its fluorescence was negligible as a result of the strong stacking of the ZnPc units in the nanoparticles in these aqueous media.^53^

The singlet oxygen generation ability of these solutions was then determined using 1,3-diphenylisobenzofuran (DPBF) as a probe, which reacts with singlet oxygen to form 1,2-dibenzoylbenzene through an unstable peroxide intermediate.^54^ The photosensitizing efficiency is reflected by the rate of consumption of DPBF upon light irradiation, as monitored spectroscopically at its absorption at 415 nm. As depicted in Figure 3c, Gal-(ZnPc*)_**2**_**-NP** in DMF could quickly consume DPBF with a rate just slightly slower than that of ZnPc*. This observation indicates that the quenching in singlet oxygen generation was not as efficient in this dimeric system as observed previously.^47,48^ Interestingly, there was no observable change in the absorbance of the nanoparticles in water or PBS, indicating that the dimer could not generate singlet oxygen under these conditions. The trend was in accordance with that observed based on the fluorescence emission (Figure 3b). The overall results demonstrate that by encapsulating the molecules of Gal-(ZnPc*)_**2**_ in nanoparticles, it can promote the molecular aggregation and the self-quenching effect, giving a fully quenched photosensitizing system.

The activation effect of β-gal on the fluorescence emission of Gal-(ZnPc*)_**2**_**-NP** was then studied in PBS with Tween 80 (0.01% v/v) at 37 °C. Since the free ZnPc* released after activation could not be completely dissolved in this aqueous medium, a trace amount of the surfactant Tween 80 was added to increase its solubility. As shown in Figure 3d, the spectrum was not significantly changed over a period of 30 h in the absence of β-gal, showing that the nanoparticles remained intact under these conditions. In contrast, the fluorescence was largely recovered upon the addition of β-gal (10 unit mL^–1^) (Figure 3e). The intensity almost reached the maximum after the treatment for 24 h. It is noteworthy that the addition of a trace amount of Tween 80 could partially relax the π–π stacking of the phthalocyanine units, as reflected by the slightly higher fluorescence intensity (Figure 3b). The time-independent fluorescence intensity suggested that the dimer remained predominantly in a nanoparticle form under these conditions. After full activation, the fluorescence intensity increased by more than 4-fold, which is larger than the difference in fluorescence intensity between Gal-(ZnPc*)_**2**_**-NP** and ZnPc* in DMF (ca. 3-fold) (Figure 3b). This observation was also consistent with a nanostructure for Gal-(ZnPc*)_**2**_**-NP** in PBS with Tween 80, which provided an additional quenching mechanism for the dimer. The disassembly of the nanoparticles after activation was also confirmed by TEM, which showed that the well-defined spherical shape of the nanoparticles became blurred (Figure S6).

The percentage of fluorescence recovery was determined at different time points by assuming that the maximum fluorescence intensity that could be recovered was the fluorescence intensity of ZnPc* at 2-fold the concentration under the same conditions. It was found that the percentage of fluorescence recovery reached about 70% after the treatment with β-gal for 30 h, while the percentage was less than 10% in the absence of β-gal (Figure 3f). To confirm that the restoration of fluorescence emission was due to the β-gal-triggered release of free ZnPc* as proposed in Figure 1, the reaction mixture of Gal-(ZnPc*)_**2**_**-NP** and β-gal after being stirred at 37 °C for 30 h was analyzed by using matrix-assisted laser desorption/ionization time-of-flight (MALDI-TOF) mass spectrometry. The spectrum clearly showed the signal of the protonated molecular ion of ZnPc* as the base peak (Figure S7). In addition, HPLC was used to analyze the reaction mixture. As shown in Figure S8, the peak at 17.2 min corresponding to Gal-(ZnPc*)_**2**_**-NP** diminished significantly, while a new peak at 15.7 min assignable to free ZnPc* was observed. The latter was also characterized by ESI mass spectrometry.

Apart from the time-dependent study, the effect of the concentration of β-gal was also investigated. Figure 3g shows the change in the fluorescence spectrum of Gal-(ZnPc*)_**2**_**-NP** (1 μM) in PBS with Tween 80 (0.01% v/v) after mixing with different concentrations of β-gal (from 0.01 to 10 unit mL^–1^) at 37 °C for 30 h. As expected, the intensity increased with the concentration of β-gal, and a linear relationship was established in the range of 0–0.05 unit mL^–1^. The detection limit was determined to be 5 × 10^–3^ unit mL^–1^, showing that the probe has high sensitivity toward β-gal.

Similarly, β-gal could also promote the singlet oxygen generation ability of Gal-(ZnPc*)_**2**_**-NP** (Figure 3h). The efficiency of the activated product obtained after the nanoparticles were treated with β-gal at 37 °C for 30 h was only slightly lower than that of ZnPc*. All of these results show that the photoactivity of Gal-(ZnPc*)_**2**_**-NP** can be remarkably restored upon the treatment with β-gal.

### In Vitro Activation by Senescence-Associated β-gal

Being encouraged by these results, we further examined the in vitro response of Gal-(ZnPc*)_**2**_**-NP** toward senescence-associated β-gal in senescent cells. The senescent-cell model was prepared according to our previously described procedure.^43^ In brief, HeLa human cervical adenocarcinoma cells were sequentially incubated with doxorubicin (50 nM) for 72 h and then in a drug-free medium for a further 24 h. The induced cellular senescence was then assessed using an X-Gal staining assay and the fluorogenic probe C_12_FDG.^55^ As shown in the X-Gal staining images in Figure S9a, the morphology of the cells was significantly changed and showed an obvious enlargement after treatment with doxorubicin. Moreover, using the probe C_12_FDG, the fluorescence intensity in the senescent cells was found to be significantly higher (by ca. 3-fold) than that in the proliferating counterpart (Figure S9b). These assays confirmed that a senescence HeLa cell model had been established, in which the intracellular β-gal level was significantly increased.

**Figure 4:**
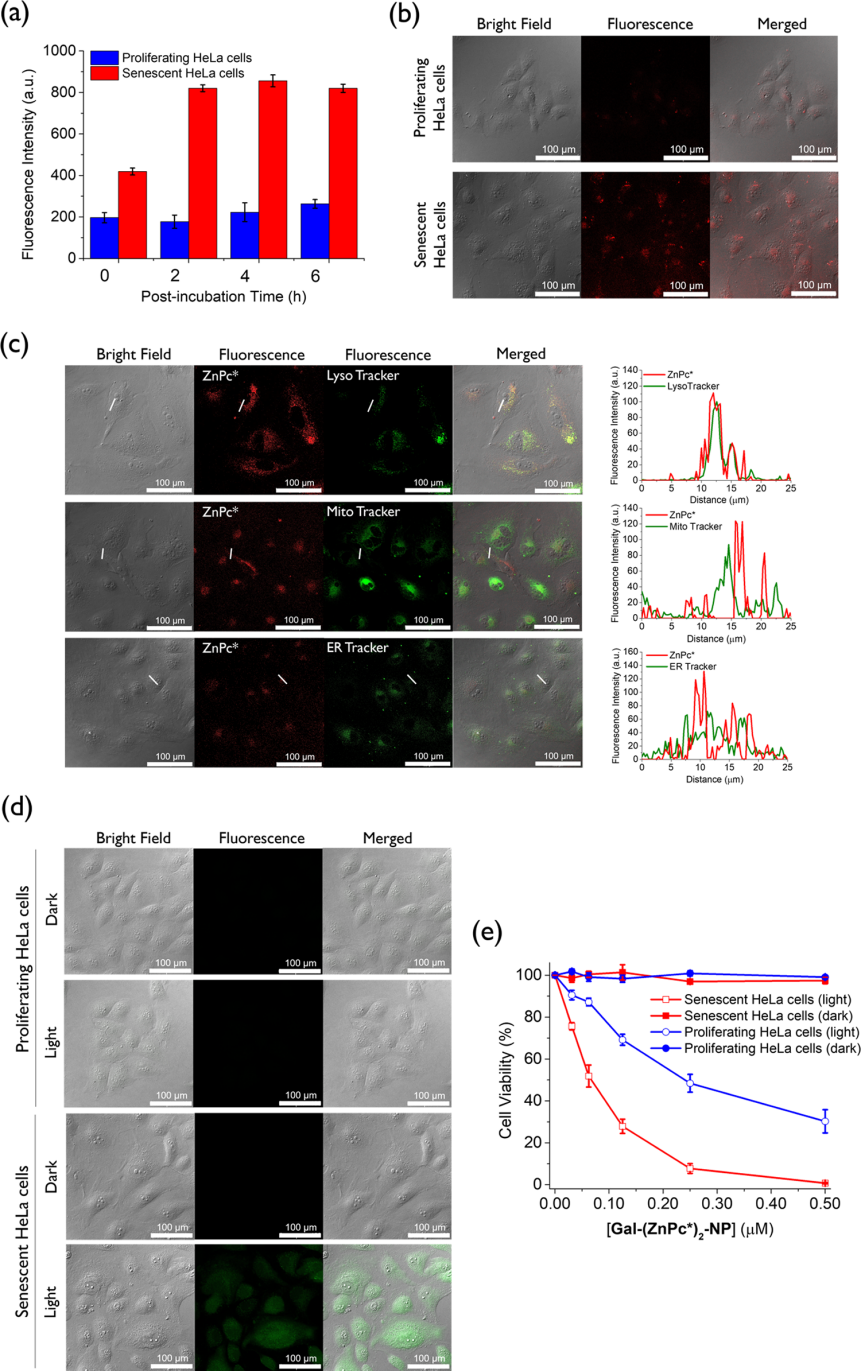
(a) Fluorescence intensities in proliferating and senescent HeLa cells after incubation with Gal-(ZnPc*)_**2**_**-NP** (2 μM) for 2 h, followed by incubation in the culture medium for different periods of time measured by flow cytometry. Data are expressed as the mean ± standard error of the mean (SEM) of three independent experiments. (b) Confocal images of proliferating and senescent HeLa cells after incubation with Gal-(ZnPc*)_**2**_**-NP** (2 μM) for 2 h and then in the culture medium for a further 2 h. (c) Visualization of the intracellular fluorescence of the activated form of Gal-(ZnPc*)_**2**_**-NP** and various subcellular trackers in senescent HeLa cells as well as the corresponding fluorescence intensity profiles. (d) Intracellular ROS as shown by the fluorescence of DCF in proliferating and senescent HeLa cells after sequential incubation with Gal-(ZnPc*)_**2**_**-NP** (0.5 μM) for 2 h, in the culture medium for a further 2 h, and then with H_2_DCFDA (10 μM) for 30 min, followed by dark or light (λ > 610 nm, fluence rate = 23 mW cm^–2^) treatment for 5 min. (e) Cytotoxicity of Gal-(ZnPc*)_**2**_**-NP** against proliferating and senescent HeLa cells for both dark and light (λ > 610 nm, fluence rate = 23 mW cm^–2^) treatment for 20 min. Data are expressed as the mean ± SEM of three independent experiments, each performed in quadruplicate.

To optimize the conditions for intracellular activation, the senescent HeLa cells were incubated with Gal-(ZnPc*)_**2**_**-NP** (2 μM) in a serum-free medium for 2 h with or without further incubation in the culture medium for 2, 4, and 6 h. The use of a serum-free medium in the first step could avoid binding between the ZnPc dimer and serum proteins. The proliferating HeLa cells without the pretreatment with doxorubicin were used as a negative control. As shown by flow cytometry, the fluorescence intensity of the senescent cells increased by 2-fold when they were postincubated for 2 h, while the intensity did not change further upon prolonged postincubation (Figure 4a). As expected, the fluorescence intensity remained low and unchanged for the proliferating HeLa cells under the same conditions. The results strongly suggest that Gal-(ZnPc*)_**2**_**-NP** is disassembled inside the senescent cells and activated by the senescence-associated β-gal therein, and these processes can be completed in about 4 h. Under these optimal incubation conditions, the fluorescence intensity of the senescent cells was about 4.5-fold higher than that of the proliferating cells. The enhancement was significantly larger than that observed using our previously reported BODIPY-based photosensitizer (3.1-fold) and the commercial probe C_12_FDG (2.5-fold),^43^ showing that this nanosystem behaved as a more efficient fluorescent probe for detecting cellular senescence. The stronger intracellular fluorescence in senescent cells caused by Gal-(ZnPc*)_**2**_**-NP** was also observed in their confocal images (Figure 4b).

The subcellular localization of Gal-(ZnPc*)_**2**_**-NP (**or strictly speaking, ZnPc* released after activation) in senescent HeLa cells was then further examined by confocal microscopy. After incubation with these nanoparticles (2 μM) for 2 h and then in the culture medium for 2 h, the cells were stained with LysoTracker Green DND-26 (2 μM), MitoTracker Green FM (0.2 μM), or ER-Tracker Green (1 μM) for 30, 15, and 15 min, respectively. The fluorescence profile of the activated species was found to overlap well with that of LysoTracker, but not the other two trackers (Figure 4c), showing that the nanoparticles exhibit a high degree of lysosomal localization, where the overproduced β-gal activates them to release ZnPc*.

In addition to the study of fluorescence recovery, the restoration of the ROS production ability of Gal-(ZnPc*)_**2**_**-NP** in senescent HeLa cells was also investigated using 2′,7′-dichlorodihydrofluorescein diacetate (H_2_DCFDA) as a probe. Upon oxidation by the intracellular ROS, it generates 2′,7′-dichlorofluorescein (DCF) as a highly emissive product that can be detected readily by confocal fluorescence microscopy.^56^ In this study, both the proliferating and senescent HeLa cells were sequentially incubated with Gal-(ZnPc*)_**2**_**-NP** (0.5 μM) for 2 h, in the culture medium for 2 h, and then with H_2_DCFDA (10 μM) for 30 min, followed by dark or light (λ > 610 nm, fluence rate = 23 mW cm^–2^) treatment for 5 min before being examined by confocal microscopy (Figure 4d). As expected, for proliferating HeLa cells, the intracellular fluorescence was negligible regardless of whether the cells had been irradiated, which could be attributed to the low intrinsic β-gal level. For senescent cells, while the fluorescence remained weak for the dark treatment group, notable fluorescence was observed for the irradiated group, demonstrating that Gal-(ZnPc*)_**2**_**-NP** is activated in senescent cells and generates ROS effectively upon light irradiation.

With this β-gal-responsive property, it was expected that Gal-(ZnPc*)_**2**_**-NP** could selectively eliminate senescent cells. To demonstrate this effect, both the proliferating and senescent HeLa cells were incubated with various concentrations of these nanoparticles for 2 h and then in the culture medium for a further 2 h, followed by dark or light (λ > 610 nm, fluence rate = 23 mW cm^–2^) treatment for 20 min. The cytotoxicity under these conditions as determined by the CellTiter-Glo luminescent cell viability assay^57^ is depicted in Figure 4e. In the absence of light irradiation, the nanoparticles were not cytotoxic to the proliferating and senescent cells. Upon light irradiation, the cell viability of proliferating HeLa cells dropped with a half-maximal inhibitory concentration (IC_50_ value) of 0.24 μM. Interestingly, the nanoparticles were much more toxic toward the senescent cells, for which the IC_50_ value was largely reduced to 0.06 μM. It is worth mentioning that for the β-gal-activatable methylene blue-based photosensitizer reported previously,^41^ the difference in photocytotoxicity was remarkable when rat glial tumor C6 cells and the β-gal-expressing *lac*Z gene-transfected counterpart were used. However, when the proliferating and palbociclib-induced senescent MDA-MB231 breast cancer cells were used, the difference was significantly reduced, and the cell viability for the latter could only drop to 60% even with a drug dose of 30 μM. In another study involving the same photosensitizer,^40^ there was a 4.5-fold difference in cell viability (ca. 90% vs 20%) against the proliferating and doxorubicin-induced senescent HeLa cells at a drug dose of 10 μM upon light irradiation. The IC_50_ value for the latter (1 μM) was much higher than that of Gal-(ZnPc*)_**2**_**-NP** (0.06 μM). These results show that for senescent cells, the β-gal expression levels depend largely on the senescence-inducing methods and the cell models, which could significantly affect the cell selectivity. The very high potency of Gal-(ZnPc*)_**2**_**-NP** may also explain that even for proliferating HeLa cells, the photocytotoxicity was not negligible.

As ZnPc* is the expected product after activation of Gal-(ZnPc*)_**2**_**-NP** by the senescence-associated β-gal, its cytotoxicity was also examined against proliferating and senescent HeLa cells under the same conditions for comparison. As shown in Figure S10, while the compound was not cytotoxic in the absence of light, it exhibited high cytotoxicity upon light irradiation. The cytotoxicity was virtually the same for both cell lines, with an IC_50_ value of 0.06 μM, which was significantly lower than that of Gal-(ZnPc*)_**2**_**-NP** against the senescent HeLa cells (0.06 or 0.12 μM with respect to the ZnPc* unit). These results are expected as ZnPc* is an “always-on” photosensitizer that does not require activation to generate cytotoxic ROS for cell killing.

## Conclusions

In summary, we have designed and synthesized a novel dimeric ZnPc conjugated with a β-galactose moiety via a self-immolative linker, i.e., Gal-(ZnPc*)_**2**_. This compound undergoes self-assembly in aqueous media, forming stable nanospheres with a hydrodynamic diameter of 68 nm, whose fluorescence emission and ROS generation are largely quenched by the exaggerated stacking of the molecules. Upon interaction with β-gal, these photoactivities can be restored through selective cleavage of the glycosidic bonds, followed by self-immolation to release the monomeric ZnPc* units. By using a senescent HeLa cell model, it has been further demonstrated that Gal-(ZnPc*)_**2**_**-NP** can be disassembled inside the cells and activated by the overproduced senescence-associated β-gal therein. The fluorescence intensity of the senescent cells is about 4.5-fold higher than that of the proliferating cells, showing that the nanosystem can serve as an efficient fluorescent probe for detecting cellular senescence. Its intracellular ROS generation ability can also be activated, enabling effective killing of the senescent cells with an IC_50_ value as low as 0.06 μM. All the results show that Gal-(ZnPc*)_**2**_**-NP** is a novel nanophotosensitizer that can be prepared readily by self-assembly without the need of any carriers and can effectively detect and eliminate senescent cells. This work also demonstrates that PDT is a promising approach for antisenescence treatment.

## Experimental Section

### General

All the reactions were performed under an atmosphere of nitrogen and monitored by thin-layer chromatography performed on Merck precoated silica gel 60 F254 plates. DMF was purified using an INERT solvent purification system. All other solvents and reagents were of reagent-grade and used as received. Chromatographic purification was performed with column chromatography on silica gel (Macherey-Nagel, 230–400 mesh). ZnPc*(50) and **1**(43) were prepared as described.

^1^H NMR spectra were recorded on a Bruker AVANCE III 500 MHz spectrometer in CDCl_3_ or DMSO-*d*_6_. Spectra were referenced internally using the residual solvent resonance (δ = 7.26 ppm for CDCl_3_ or 2.50 ppm for DMSO-*d*_6_) relative to SiMe_4_. MALDI-TOF mass spectra were recorded on a Bruker Autoflex Speed MALDI-TOF mass spectrometer. High-resolution ESI mass spectra were recorded on a Thermo Finnigan MAT 95 XL mass spectrometer. Electronic absorption and steady-state fluorescence spectra were taken on a Cary 5G UV–vis-NIR spectrophotometer and a HORIBA FluoroMax-4 spectrofluorometer, respectively. TEM images were taken using a FEI Tecnai G2 Spirit transmission electron microscope operated at a 120 kV acceleration voltage. The hydrodynamic diameters of the nanoparticles were measured using a DelsaMax Pro analyzer.

Reverse-phase HPLC analysis was performed on an XBridge BEH300 C18 column (5 μm, 4.6 × 150 mm) at a flow rate of 1 mL min^–1^ using a Waters system equipped with a Waters 1525 binary pump and a Waters 2998 photodiode array detector. The solvents used were of HPLC-grade. The conditions were set as follows: solvent A = 0.1% trifluoroacetic acid (TFA) and 5% DMSO in acetonitrile, and solvent B = 0.1% TFA in deionized water. Elution gradient: 50% A + 50% B in the first 5 min; changed to 100% A + 0% B in 5 min; maintained under this condition for 20 min; changed to 50% A + 50% B in 5 min; maintained under this condition for a further 25 min. Mass spectra were recorded with a Waters single quadrupole detector 2. The purity of the end product Gal-(ZnPc*)_**2**_ was found to be >95% by HPLC analysis.

### Preparation of **2**

A mixture of ZnPc* (50 mg, 56 μmol), **1** (32 mg, 28 μmol), and Et_3_N (39 μL, 0.28 mmol) in DMF (5 mL) was stirred at room temperature overnight. The solvent was then evaporated under reduced pressure, and the residue was purified by column chromatography on silica gel with CHCl_3_/MeOH (15:1 v/v) as the eluent to afford **2** (27 mg, 36%) as a green solid. ^1^H NMR (500 MHz, CDCl_3_ with a trace amount of pyridine-d_5_): δ 9.44 and 9.37 (two br s, 2 H, Pc-H_α_), 9.15 (br s, 6 H, Pc-H_α_), 8.90 (br s, 4 H, Pc-H_α_), 7.89–8.23 (m, 11 H, Pc-H_β_ and ArH), 7.74 (s, 1 H, ArH), 7.70 (s, 1 H, ArH), 7.47–7.57 (m, 1 H, Pc-H_β_), 7.30–7.40 (m, 1 H, Pc-H_β_), 7.15–7.18 (m, 2 H, ArH), 6.95–7.12 (m, 4 H, Pc-H_β_), 5.78 (br s, 2 H, NH), 5.46–5.52 (m, 1 H), 5.42 (br s, 1 H), 4.99–5.10 (m, 4 H), 4.84–4.97 (m, 6 H), 4.72–4.76 (m, 6 H), 4.51–4.53 (m, 1 H), 4.34–4.40 (m, 6 H), 4.14–4.18 (m, 1 H), 4.10 (t, *J* = 4.5 Hz, 4 H), 4.03 (br s, 3 H), 3.96–3.99 (m, 1 H), 3.84 (t, *J* = 4.5 Hz, 4 H), 3.76 (br s, 3 H), 3.70 (t, *J* = 4.5 Hz, 4 H), 3.58 (br s, 3 H), 3.54 (t, *J* = 4.5 Hz, 4 H), 3.46–3.49 (m, 1 H), 3.42 (br s, 2 H), 3.35 (s, 6 H), 3.34 (s, 3 H), 2.86–3.07 (m, 6 H, NCH_3_), 2.14–2.15 (m, 3 H, OAc), 2.11 (s, 3 H, OAc), 2.00 (s, 3 H, OAc), 1.94–1.97 (m, 3 H, OAc). HRMS (ESI): *m*/*z* calcd for C_128_H_128_N_21_NaO_34_Zn_2_ [M+H+Na]^2+^: 1328.8705, found 1328.8703.

### Preparation of Gal-(ZnPc*)_2_

A mixture of **2** (20 mg, 7.6 μmol) and NaOMe (2.2 mg, 0.04 mmol) in CHCl_3_/MeOH (4:1 v/v, 10 mL) was stirred at room temperature overnight. The solvent was then evaporated under reduced pressure, and the residue was purified by column chromatography on silica gel with CHCl_3_/MeOH (8:1 v/v) as the eluent to afford Gal-(ZnPc*)_**2**_ (15 mg, 82%) as a green solid. ^1^H NMR (500 MHz, DMSO-*d*_6_ with a trace amount of pyridine-d_5_): δ 9.42 and 9.36 (br s, 1 H, Pc-H_α_), 8.98–9.03 (m, 6 H, Pc-H_α_), 8.72–8.79 (m, 4 H, Pc-H_α_), 8.25 (br s, 1 H, Pc-H_α_), 7.83–8.02 (m, 10 H, Pc-H_β_), 7.59–7.61 (m, 2 H, Pc-H_β_), 7.39 (d, *J* = 8.5 Hz, 1 H, ArH), 7.27–7.31 (m, 2 H, ArH), 7.20 (s, 2 H, ArH), 7.10–7.14 (m, 4 H, Pc-H_β_), 5.17 (d, *J* = 4.5 Hz, 1 H), 5.05 (br s, 2 H), 5.01 (d, *J* = 7.5 Hz, 1 H), 4.87–4.93 (m, 4 H), 4.67 (br s, 6 H), 4.60 (br s, 1 H), 4.38 (br s, 1 H), 4.24 (br s, 6 H), 4.02 (br s, 1 H), 3.95 (br s, 6 H), 3.68–3.70 (m, 8 H), 3.46–3.61 (m, 12 H), 3.38–3.40 (m, 12 H), 3.19 (br s, 3 H), 3.17 (s, 3 H), 3.14–3.15 (m, 1 H), 3.04–3.08 (m, 2 H), 2.80–2.94 (m, 5 H), 2.64 (m, 1 H), 2.36 (m, 1 H), 2.28 (br s, 2 H). HRMS (ESI): *m*/*z* calcd for C_120_H_121_N_21_O_30_Zn_2_ [M+2H]^2+^: 1233.8582, found 1233.8576.

### Preparation of Gal-(ZnPc*)_2_-NP

A stock solution of Gal-(ZnPc*)_**2**_ in DMSO (0.4 mM) was prepared by dissolving 1 mg of the compound in 1 mL of DMSO. An aliquot (100 μL) of the stock solution was then added dropwise into water (3.9 mL) with sonication. The resulting mixture was sonicated for 2 h to form the self-assembled nanoparticles Gal-(ZnPc*)_**2**_**-NP** (10 μM). A portion (900 μL) of this suspension was further diluted with 10X serum-free RPMI 1640 medium (100 μL) to give a stock solution of the nanoparticles (9 μM) for the following studies.

### Measurement of Singlet Oxygen Generation

A solution of DPBF (30 μM) and Gal-(ZnPc*)_**2**_**-NP** (1 μM in water, PBS, or DMF) or ZnPc* (2 μM in DMF) was irradiated with light from a 100 W halogen lamp after being passed through a water tank for cooling and a color filter with a cut-on wavelength of 610 nm (Newport). For the enzymatic activation, Gal-(ZnPc*)_**2**_**-NP** (1 μM) was treated with β-gal (10 unit mL^–1^) in PBS with Tween 80 (0.01% v/v) at 37 °C for 30 h before DPBF (30 μM) was added. The resulting solution was then irradiated, as described above. The absorbance of DPBF’s absorption at 415 nm was monitored along with the irradiation time. The results were compared with those for Gal-(ZnPc*)_**2**_**-NP** without the pretreatment with β-gal.

### Cell Line and Culture Conditions

HeLa cells (ATCC, no. CCL-2) were maintained in RPMI 1640 medium (Invitrogen, cat. no. 23400-021) supplemented with fetal bovine serum (10%) and penicillin-streptomycin (100 unit mL^–1^ and 100 μg mL^–1^, respectively). They were grown at 37 °C in a humidified 5% CO_2_ atmosphere.

### Confocal Fluorescence Microscopic Studies

Approximately 1 × 10^4^ HeLa cells in RPMI 1640 medium (2 mL) were seeded on a confocal dish and incubated overnight at 37 °C in a humidified 5% CO_2_ atmosphere. After removal of the medium, the cells were rinsed with PBS (1 mL) and incubated in the culture medium containing doxorubicin (50 nM) for 72 h. The cells were rinsed with PBS (1 mL) twice and then incubated in the culture medium for a further 24 h. After being rinsed with PBS, the senescent cells were used for the following study. For the proliferating HeLa cells, approximately 1 × 10^5^ cells in RPMI 1640 medium (2 mL) were seeded on a confocal dish and incubated overnight at 37 °C in a humidified 5% CO_2_ atmosphere. The number of cells used for the preparation of senescent cells was lowered by 1 order of magnitude as the number would grow during the 96 h pretreatment, and the senescent cells generally show an enlarged morphology that would make the cells pack too closely if the cell number is too large. Both the senescent and proliferating HeLa cells were incubated with Gal-(ZnPc*)_**2**_**-NP** (2 μM) in a serum-free medium at 37 °C for 2 h. After being rinsed with PBS twice, the cells were further incubated in the culture medium for 2 h. For the staining with C_12_FDG, the cells were incubated with C_12_FDG (25 μM) in the culture medium for 35 min. The solutions were then removed, and the cells were rinsed with PBS twice before being examined with a Leica TCS SP8 high-speed confocal microscope equipped with two lasers at 488 and 638 nm. ZnPc* was excited at 638 nm, and its fluorescence was monitored at 650–750 nm. C_12_FDG was excited at 488 nm, and its fluorescence was monitored at 500–600 nm. The images were digitized and analyzed using a Leica Application Suite X software.

### Flow Cytometric Studies

Approximately 1 × 10^4^ HeLa cells per well in RPMI 1640 medium (2 mL) were seeded on a 6-well plate and incubated overnight at 37 °C in a humidified 5% CO_2_ atmosphere. After removal of the medium, the cells were rinsed with PBS (1 mL) and incubated with doxorubicin (50 nM) in the culture medium for 72 h. The cells were rinsed with PBS (1 mL) twice and then incubated in the culture medium for a further 24 h. After being rinsed with PBS, the senescent cells were used for the following study. For the proliferating HeLa cells, approximately 1 × 10^5^ HeLa cells in RPMI 1640 medium (2 mL) were seeded on a confocal dish and incubated overnight at 37 °C in a humidified 5% CO_2_ atmosphere. Both the senescent and proliferating HeLa cells were incubated with Gal-(ZnPc*)_**2**_**-NP** (2 μM) in a serum-free medium at 37 °C for 2 h. After being rinsed with PBS twice, the cells were further incubated in the culture medium for 2 h. For the staining with C_12_FDG, the cells were incubated with C_12_FDG (25 μM) in the culture medium for 35 min. The solutions were then removed, and the cells were rinsed with PBS twice and then harvested with 0.25% trypsin-ethylenediaminetetraacetic acid (Invitrogen, 0.2 mL) for 5 min. The activity of trypsin was quenched with a serum-containing medium (0.5 mL), and the mixture was centrifuged at 1500 rpm for 3 min at room temperature. The pellet was washed with PBS (1 mL) and then subjected to centrifugation. The cells were suspended in PBS (1 mL), and the intracellular fluorescence intensities were measured using a BD FACSVerse flow cytometer (Becton Dickinson) with 10^4^ cells counted in each sample. ZnPc* was excited by an argon laser at 640 nm, and the emitted fluorescence was monitored at 720–840 nm. C_12_FDG was excited by an argon laser at 488 nm, and the emitted fluorescence was monitored at 500–600 nm. The data collected were analyzed using the BD FACSuite. All experiments were performed in triplicate.

### Study of Subcellular Localization

Senescent HeLa cells were incubated with Gal-(ZnPc*)_**2**_**-NP** (2 μM) in a serum-free medium at 37 °C for 2 h, followed by incubation in the culture medium for 2 h, as described above. After being rinsed with PBS twice, the cells were stained with LysoTracker Green DND-26 (Thermo Fisher Scientific Inc., L7526) (2 μM), MitoTracker Green FM (Thermo Fisher Scientific Inc., M7514) (0.2 μM), or ER-Tracker Green (Thermo Fisher Scientific Inc., E34251) (1 μM) in a serum-free medium at 37 °C for 30, 15, or 15 min, respectively. The solutions were then removed, and the cells were rinsed with PBS twice before being examined with a Leica TCS SP8 high-speed confocal microscope equipped with a 488 nm laser and a 638 nm laser. All the trackers were excited at 488 nm, and their fluorescence was monitored at 500–570 nm, while ZnPc* was excited at 638 nm, and its fluorescence was monitored at 650–750 nm. The images were digitized and analyzed using Leica Application Suite X software.

### Study of Intracellular ROS Generation

Senescent or proliferating HeLa cells were incubated with Gal-(ZnPc*)_**2**_**-NP** (0.5 μM) in a serum-free medium at 37 °C for 2 h, followed by incubation in the culture medium for 2 h, as described above. After being rinsed with PBS twice, the cells were incubated with H_2_DCFDA in PBS (10 μM, 1 mL) at 37 °C for 30 min. The cells were rinsed and refilled with PBS before being irradiated at ambient temperature. The light source consisted of a 300 W halogen lamp, a water tank for cooling, and a color glass filter (Newport) cut-on at λ = 610 nm. The fluence rate (λ > 610 nm) was 23 mW cm^–2^. Irradiation for 5 min led to a total fluence of 7 J cm^–2^. After irradiation, the cells were examined with a Leica TCS SP8 high-speed confocal microscope equipped with a 488 nm laser. The fluorescent product after the oxidation of H_2_DCFDA by ROS, namely DCF, was excited at 488 nm, and its fluorescence was monitored at 500–550 nm. The images were digitized and analyzed using Leica Application Suite X software. The results were compared with those without light irradiation.

### Study of Dark and Photocytotoxicity

Approximately 1 × 10^3^ HeLa cells per well in RPMI 1640 medium were inoculated in 96-well plates and incubated overnight at 37 °C in a humidified 5% CO_2_ atmosphere. After removal of the medium, the cells were rinsed with PBS and incubated with doxorubicin (50 nM) in the culture medium for 72 h. The cells were rinsed with PBS twice and incubated in a fresh medium for a further 24 h. After being rinsed with PBS, the senescent cells were used for the following study. For the proliferating HeLa cells, approximately 1 × 10^4^ HeLa cells per well in RPMI 1640 medium were inoculated in 96-well plates and incubated overnight at 37 °C in a humidified 5% CO_2_ atmosphere. A stock solution of Gal-(ZnPc*)_**2**_**-NP** (9 μM) was prepared as described above, and the solution was then diluted with a serum-free medium to different concentrations. The cells, after being rinsed with PBS twice, were incubated with 100 μL of Gal-(ZnPc*)_**2**_**-NP** solutions at 37 °C for 2 h under 5% CO_2_. After being rinsed with PBS twice, the cells were further incubated in a serum-free medium for 2 h. The cells were then rinsed again with PBS and refed with 100 μL of the culture medium before being irradiated at ambient temperature using the aforementioned light source. Irradiation for 20 min led to a total fluence of 28 J cm^–2^. Cell viability was determined by a CellTiter-Glo luminescent cell viability assay.^57^ After irradiation, the cells were incubated at 37 °C under 5% CO_2_ overnight. A CellTiter-Glo reagent (Promega) solution (100 μL) was added to each well, and the solutions in all wells were mixed on an orbital shaker to induce cell lysis. The plate was incubated at room temperature for 10 min to stabilize the luminescence signal. The luminescence signal of each well on the plate was taken with a microplate reader (Tecan Spark 10M) at ambient temperature. The average intensity of the blank wells, which did not contain cells, was subtracted from the readings of the other wells. The cell viability was then determined by the equation:
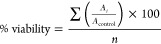
where *A_i_* is the luminescence intensity of the *i*th datum (*i* = 1, 2, ..., *n*), *A*_control_ is the average luminescence intensity of the control wells in which the compound was absent, and *n* (=4) is the number of data points. The cytotoxicity of ZnPc* was studied by using the same procedure.

## Supporting Information Available

^1^H NMR and ESI mass spectra of **2** and **Gal-(ZnPc*)_2_**, HPLC chromatogram of **Gal-(ZnPc*)_2_**, TEM images of **Gal-(ZnPc*)_2_-NP** before and after the treatment with β-gal, MALDI-TOF mass spectrum and HPLC chromatogram of the reaction mixture of **Gal-(ZnPc*)_2_-NP** and β-gal, study of the cellular senescence of HeLa cells, and cytotoxicity of **ZnPc*** (PDF)
Molecular formula strings (CSV)

## Abbreviations

β-gal: β-galactosidase
BODIPY: boron dipyrromethene
DCF: 2′,7′-dichlorofluorescein
DLS: dynamic light scattering
DMF: *N*,*N*-dimethylformamide
DMSO: dimethyl sulfoxide
DPBF: 1,3-diphenylisobenzofuran
ESI: electrospray ionization
H_2_DCFDA: 2′,7′-dichlorodihydrofluorescein diacetate
HPLC: high-performance liquid chromatography
IC_50_: half-maximal inhibitory concentration
MALDI-TOF: matrix-assisted laser desorption/ionization time-of-flight
PBS: phosphate-buffered saline
PDI: polydispersity index
PDT: photodynamic therapy
ROS: reactive oxygen species
RPMI: Roswell Park Memorial Institute
SEM: standard error of the mean
TEM: transmission electron microscopy
TFA: trifluoroacetic acid
ZnPc: zinc(II) phthalocyanine
