# Supplementary material for: Detection and Elimination of Senescent Cells with a Self-Assembled Senescence-Associated β-Galactosidase-Activatable Nanophotosensitizer
Source: J Med Chem. 2023 Dec 19;67(1):234–44. doi: 10.1021/acs.jmedchem.3c01306 (PMC10788907; doi:10.1021/acs.jmedchem.3c01306)
Supplement: Supplementary file 1 — jm3c01306_si_001.pdf [file jm3c01306_si_001.pdf]

Supporting Information

Detection and Elimination of Senescent Cells with a Self-Assembled Senescence-Associated β-Galactosidase-Activatable Nanophotosensitizer

Jacky C. H. Chu,[†,#] Junlong Xiong,[†,‡,#] Clarence T. T. Wong,[†,¶] Shuai Wang,[†] Dick Yan Tam,[†] Alba García-Fernández,[§,‖,⊥] Ramón Martínez-Máñez,[§,‖,⊥,Γ] and Dennis K. P. Ng[†,*]

[†] *Department of Chemistry, The Chinese University of Hong Kong, Shatin, N.T., Hong Kong, China. E-mail: dkpn@cuhk.edu.hk*

[‡] *Department of Pharmacy, The Affiliated Luohu Hospital of Shenzhen University, Shenzhen University, Shenzhen 518001, China*

[¶] *Department of Applied Biology and Chemical Technology, The Hong Kong Polytechnic University, Kowloon, Hong Kong, China*

[§] *Instituto Interuniversitario de Investigación de Reconocimiento, Molecular y Desarrollo Tecnológico, Universitat Politècnica de València, Universitat de València, Valencia 46022, Spain*

[‖] *CIBER de Bioingeniería, Biomateriales y Nanomedicina, Instituto de Salud Carlos III, Madrid 28029, Spain*

[⊥] *Unidad Mixta UPV-CIPF de Investigación en Mecanismos de Enfermedades y Nanomedicina, Universitat Politècnica de València, Centro de Investigación Príncipe Felipe, Valencia 46012, Spain*

Γ *Unidad Mixta de Investigación en Nanomedicina y Sensores, Universitat Politècnica e València, Instituto de Investigación Sanitaria La Fe (IIS La Fe), Valencia 46026, Spain*

# *These authors contributed equally to this work*

Contents

Figure S1 ^{1}H NMR spectrum of **2** in $CDCl_3$ with a trace amount of pyridine-d_5.

Figure S2 ^{1}H NMR spectrum of **Gal-(ZnPc*)$_2$** in DMSO-d_6 with a trace amount of pyridine-d_5.

Figure S3 ESI mass spectrum of **2** and the enlarged experimental (lower left) and simulated (lower right) isotopic patterns of the $[M+H+Na]^{2+}$ species.

Figure S4 ESI mass spectrum of **Gal-(ZnPc*)$_2$** and the enlarged experimental (lower left) and simulated (lower right) isotopic patterns of the $[M+2H]^{2+}$ species.

Figure S5 HPLC chromatogram of **Gal-(ZnPc*)$_2$**.

Figure S6 TEM images of **Gal-(ZnPc*)$_2$-NP** (a) before and (b) after the treatment with β-gal (10 unit mL^{-1}) in PBS with Tween 80 (0.01% v/v) at 37 °C for 30 h.

Figure S7 MALDI-TOF mass spectrum of the reaction mixture obtained after treating **Gal-(ZnPc*)$_2$-NP** (1 μM) with β-gal (10 unit mL^{-1}) in PBS with Tween 80 (0.01% v/v) at 37 °C for 30 h.

Figure S8 (a) HPLC chromatograms of **Gal-(ZnPc*)$_2$-NP**, the reaction mixture obtained after treating **Gal-(ZnPc*)$_2$-NP** (1 μM) with β-gal (10 unit mL^{-1}) in PBS with Tween 80 (0.01% v/v) at 37 °C for 30 h, and **ZnPc***. (b) ESI mass spectrum of the fraction with a retention time of 15.7 min.

Figure S9 (a) X-Gal staining images of HeLa cells with or without the pre-treatment with doxorubicin (50 nM) for 3 days. (b) Bright field, fluorescence, and the merged images of the proliferating and senescent HeLa cells after further incubation with C_{12}FDG (25 μM) for 35 min, and comparison of the corresponding intracellular fluorescence intensities as determined by flow cytometry.

Figure S10 Cytotoxicity of **ZnPc*** against proliferating and senescent HeLa cells in the absence and presence of light irradiation ($\lambda > 610$ nm, fluence rate = 23 mW cm^{-2}) for 20 min.

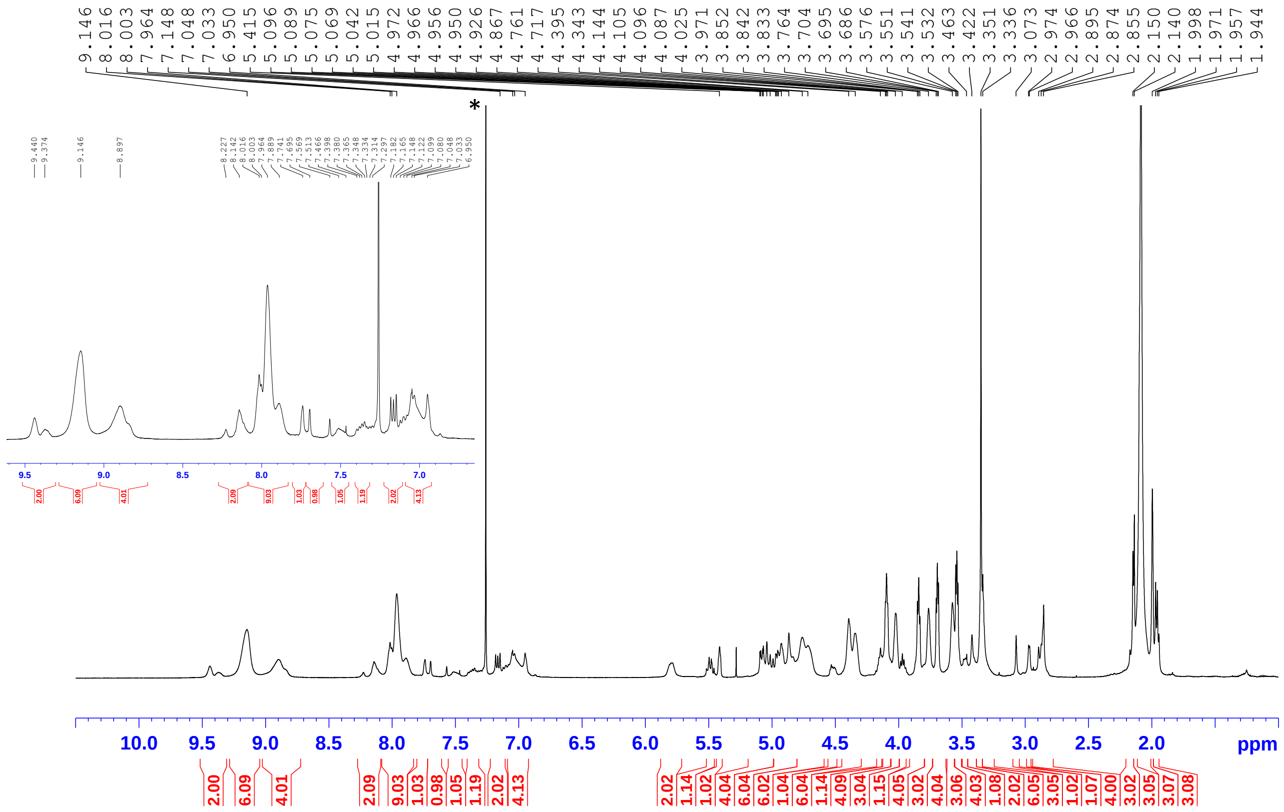

Figure S1. ^{1}H NMR spectrum of **2** in $CDCl_3$ with a trace amount of pyridine-d_5.

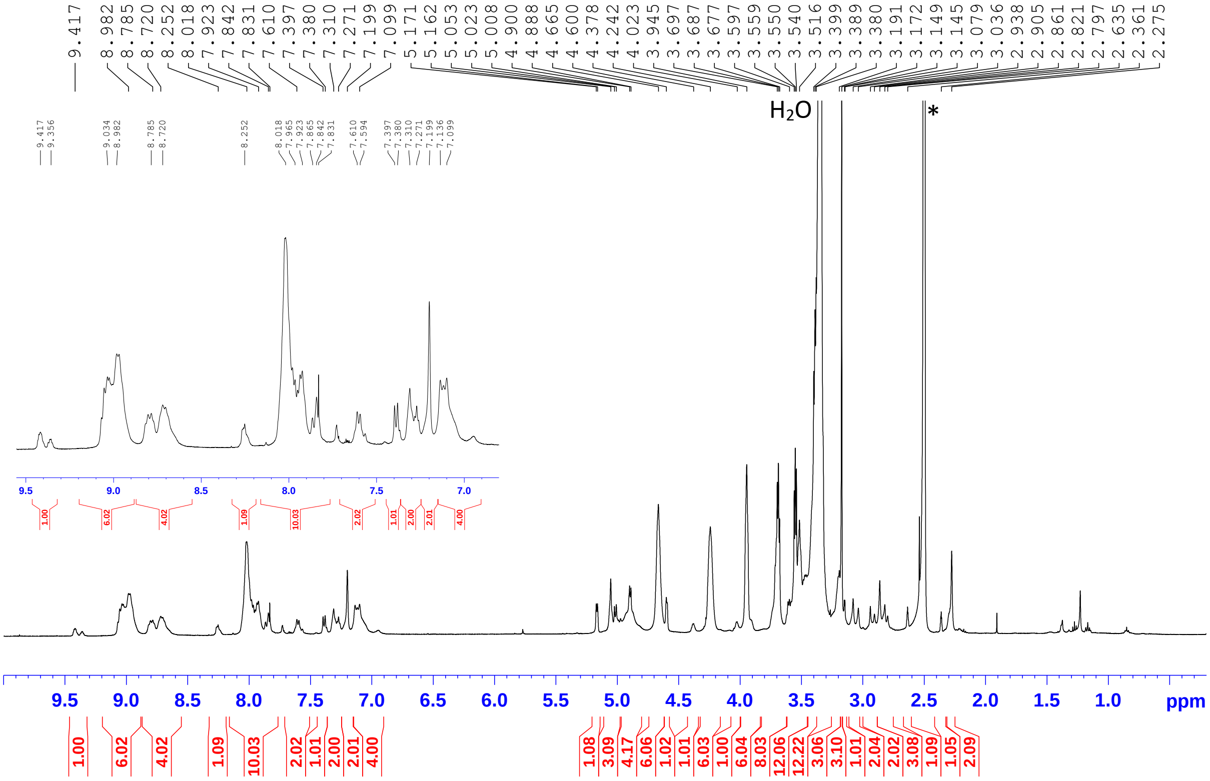

Figure S2. ^{1}H NMR spectrum of **Gal-(ZnPc*)**$_2$ in DMSO-d$_6$ with a trace amount of pyridine-d$_5$.

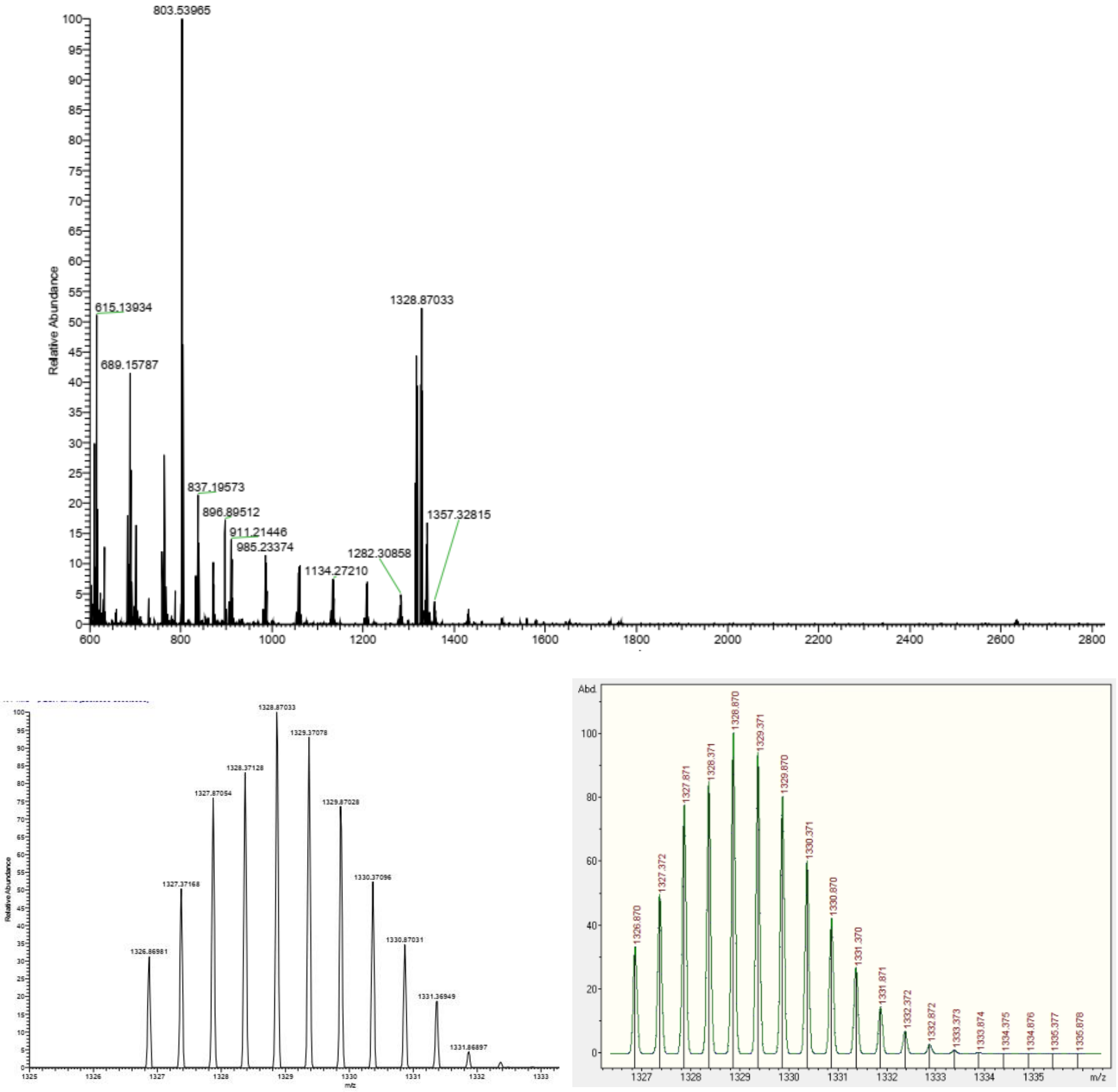

Figure S3. ESI mass spectrum of **2** and the enlarged experimental (lower left) and simulated (lower right) isotopic patterns of the $[M+H+Na]^{2+}$ species.

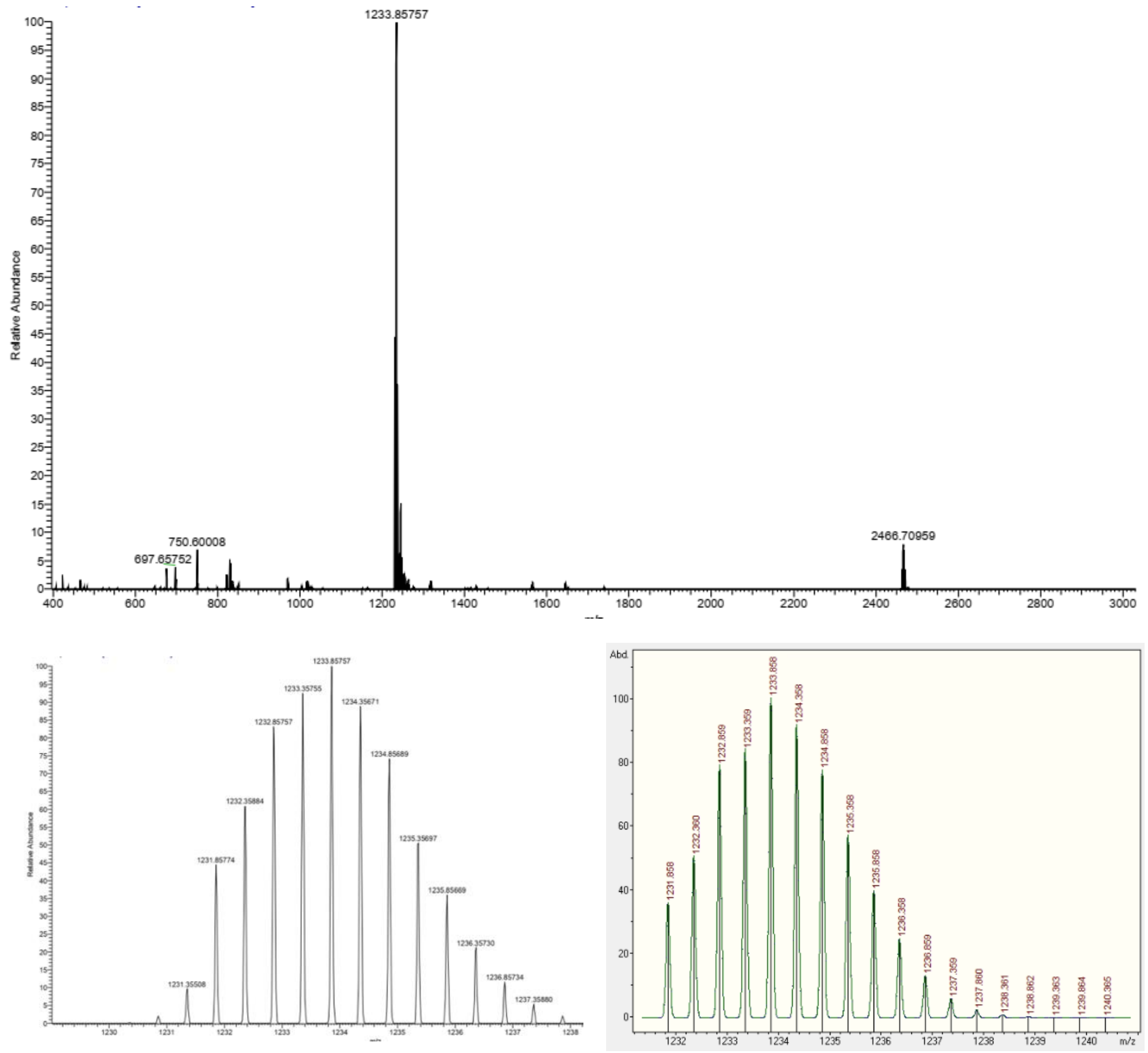

Figure S4. ESI mass spectrum of **Gal-(ZnPc*)$_2$** and the enlarged experimental (lower left) and simulated (lower right) isotopic patterns of the $[M+2H]^{2+}$ species.

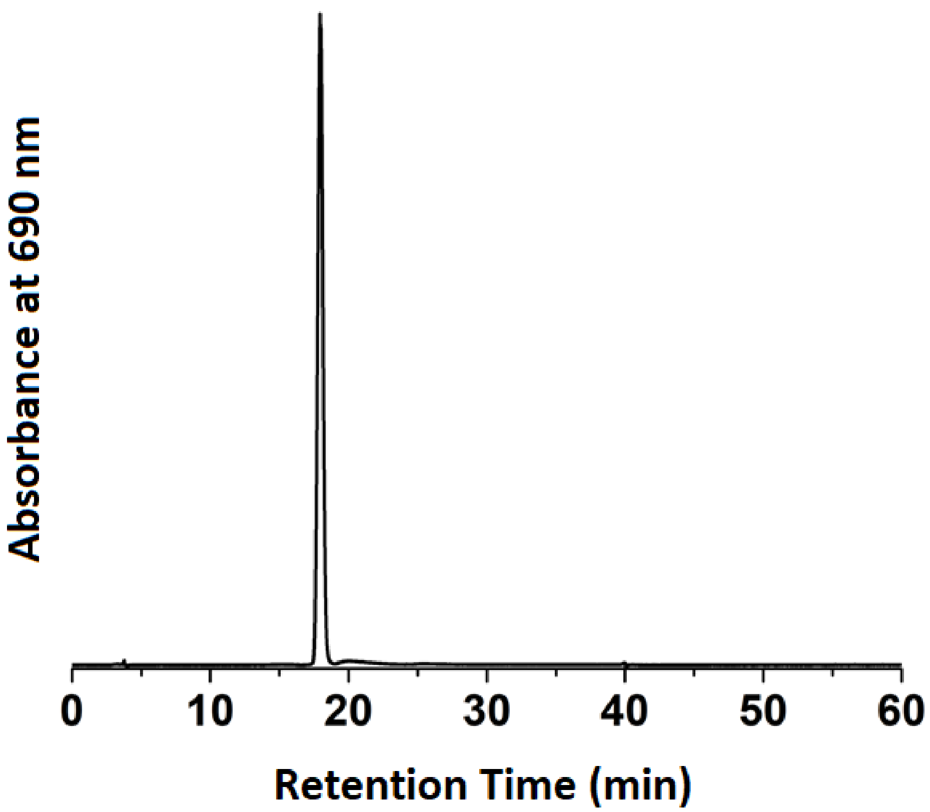

Figure S5. HPLC chromatogram of **Gal-(ZnPc*)$_2$**.

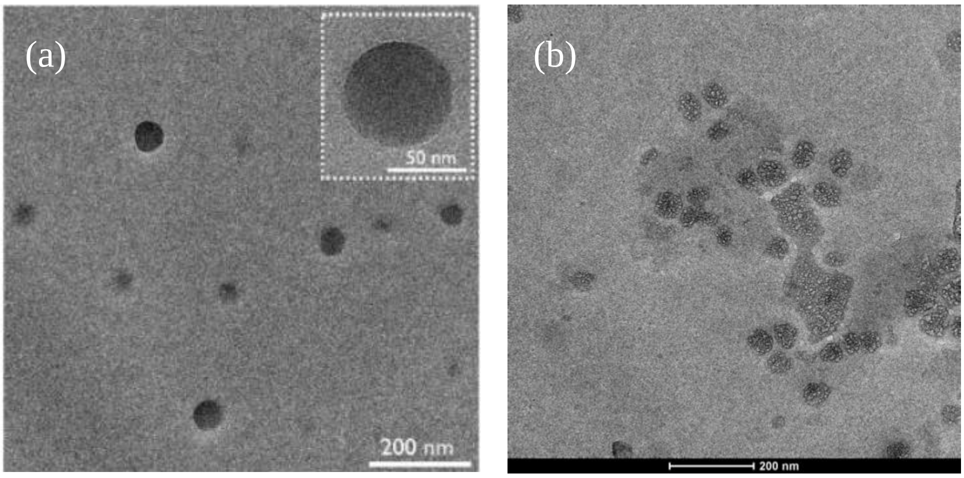

Figure S6. TEM images of **Gal-(ZnPc*)$_2$-NP** (a) before and (b) after the treatment with β-gal (10 unit mL^{-1}) in PBS with Tween 80 (0.01% v/v) at 37 ^{o}C for 30 h.

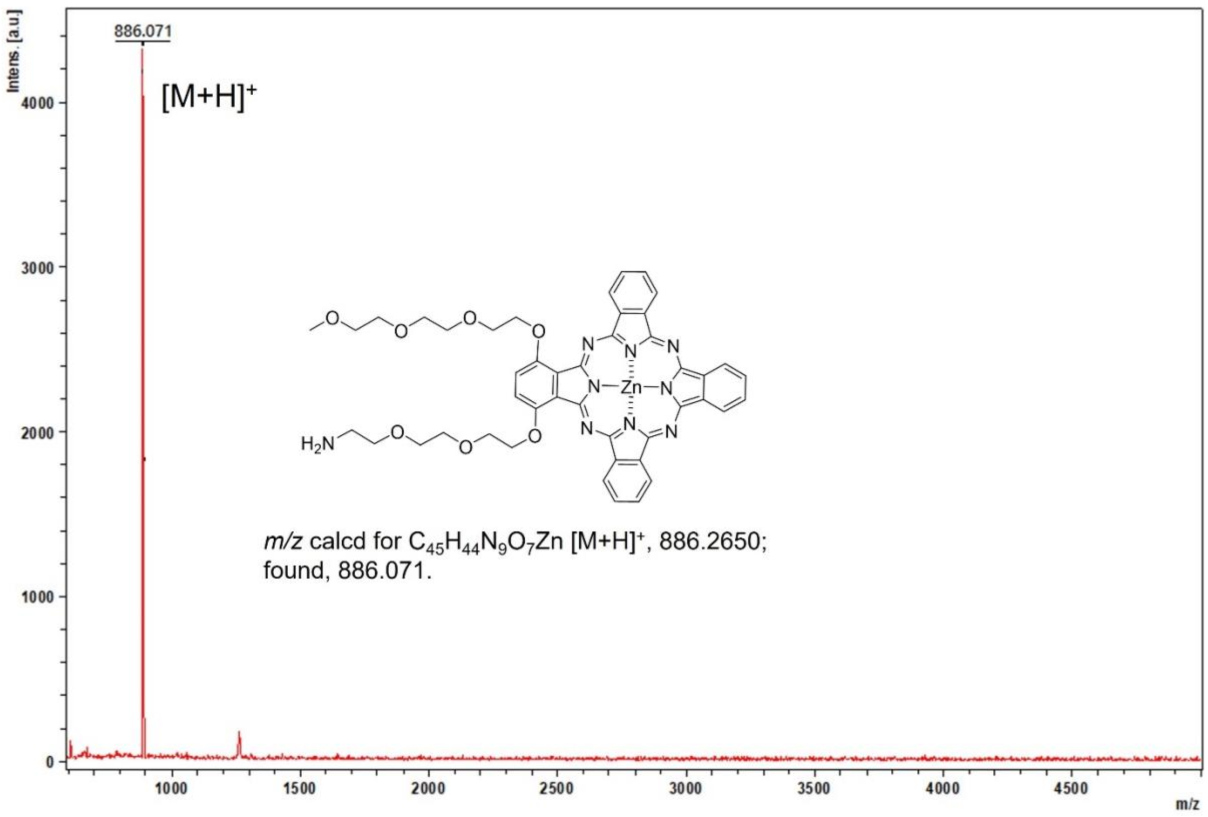

Figure S7. MALDI-TOF mass spectrum of the reaction mixture obtained after treating **Gal-(ZnPc*)$_2$-NP** (1 µM) with β-gal (10 unit mL^{-1}) in PBS with Tween 80 (0.01% v/v) at 37 ^{o}C for 30 h.

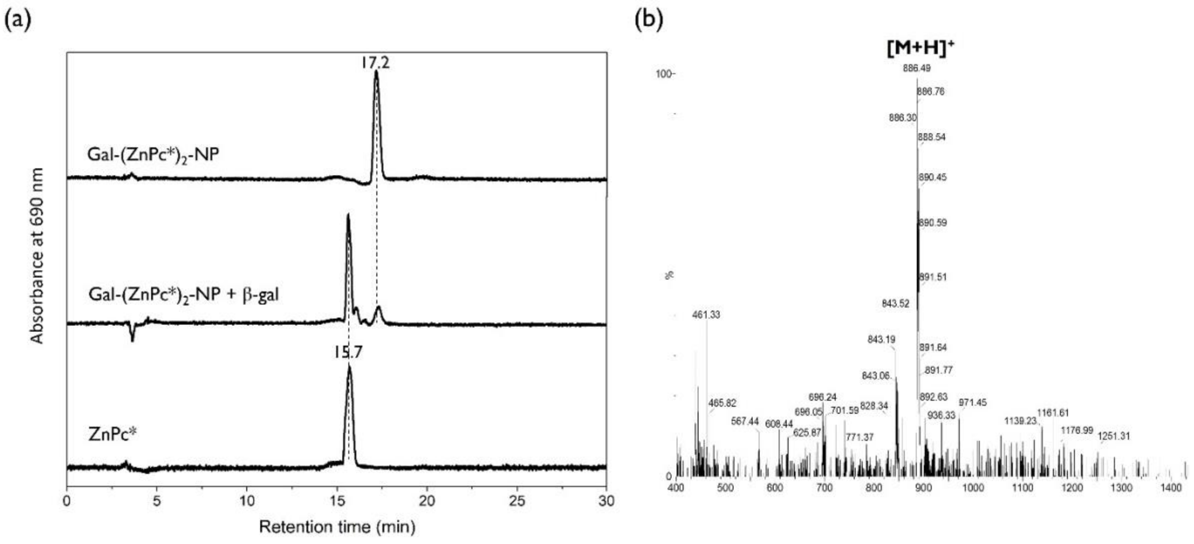

Figure S8. (a) HPLC chromatograms of **Gal-(ZnPc*)$_2$-NP**, the reaction mixture obtained after treating **Gal-(ZnPc*)$_2$-NP** (1 µM) with β-gal (10 unit mL^{-1}) in PBS with Tween 80 (0.01% v/v) at 37 ^{o}C for 30 h, and **ZnPc***. (b) ESI mass spectrum of the fraction with a retention time of 15.7 min.

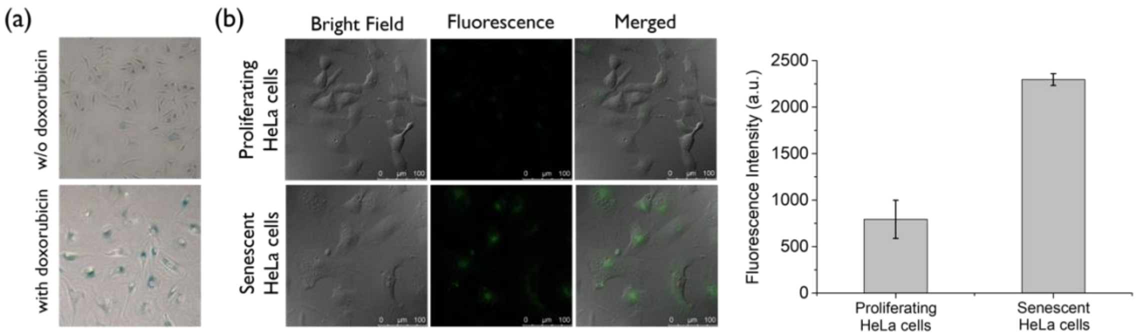

Figure S9. (a) X-Gal staining images of HeLa cells with or without the pre-treatment with doxorubicin (50 nM) for 3 days. (b) Bright field, fluorescence, and the merged images of the proliferating and senescent HeLa cells after further incubation with C_{12}FDG (25 µM) for 35 min, and comparison of the corresponding intracellular fluorescence intensities as determined by flow cytometry. Data are reported as the mean ± standard error of the mean of three independent experiments.

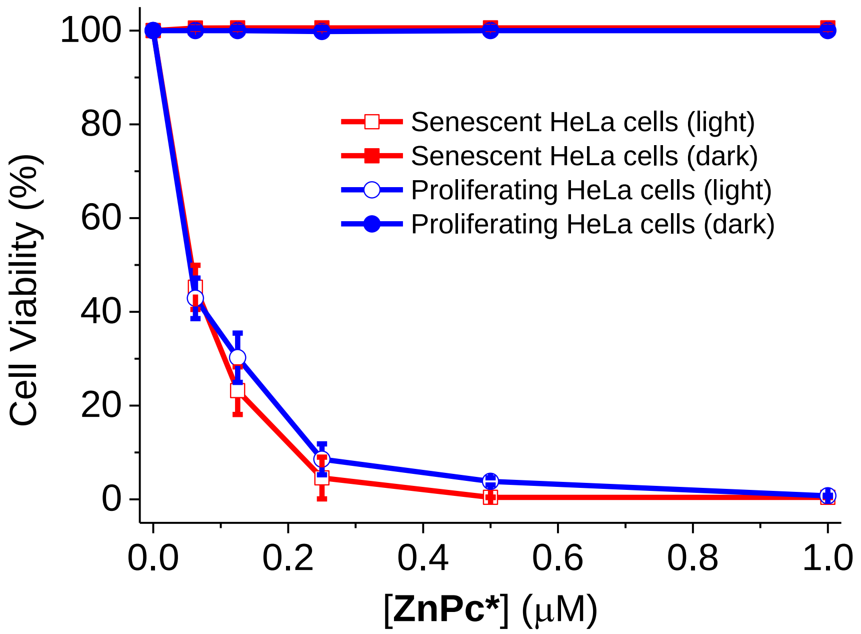

Figure S10. Cytotoxicity of **ZnPc*** against proliferating and senescent HeLa cells in the absence and presence of light irradiation ($\lambda > 610$ nm, fluence rate = 23 mW cm^{-2}) for 20 min. Data are reported as the mean ± SEM of three independent experiments, each performed in quadruplicate.
